# Combined Effect of Modified Atmosphere Packaging and UV-C Radiation on Pathogens Reduction, Biogenic Amines, and Shelf Life of Refrigerated Tilapia (*Oreochromis niloticus*) Fillets

**DOI:** 10.3390/molecules25143222

**Published:** 2020-07-15

**Authors:** César A. Lázaro, Maria Lúcia G. Monteiro, Carlos A. Conte-Junior

**Affiliations:** 1 Departmento de Salud Animaly Salud Pública, Facultad de Medicina Veterinaria, Universidad Nacional Mayor de San Marcos (UNMSM), San Borja, Lima 15021, Peru; clazarod@unmsm.edu.pe; 2 Departmento de Bioquímica, Instituto de Química, Universidade Federal do Rio de Janeiro (UFRJ), Rio de Janeiro, Rio de Janeiro 21941-909, Brazil; conte@iq.ufrj.br; 3 Núcleo de Análise de Alimentos (NAL-LADETEC), Universidade Federal do Rio de Janeiro (UFRJ), Rio de Janeiro 21941-598, Brazil; 4 Departamento de Tecnologia de Alimentos, Universidade Federal Fluminense (UFF), Niterói, Rio de Janeiro 24220-000, Brazil; 5 Programa de Pós-graduação em Vigilância Sanitária, Instituto Nacional de Controle de Qualidade em Saúde, Fundação Oswaldo Cruz (FIOCRUZ), Rio de Janeiro 21040-900, Brazil

**Keywords:** freshwater fish, hurdle technology, *Salmonella typhimurium*, *Escherichia coli* O157:H7, non-thermal preservation, oxidative stability

## Abstract

This study investigated the isolated effect of modified atmosphere packaging (MAP; 50% CO_2_ and 50% N_2_) and ultraviolet radiation (UV; 0.30 J/cm^2^) as well as their combined (MAP/UV) effect on reduction of *Salmonella typhimurium* and *Escherichia coli* O157:H7, biogenic amines (BA), and on shelf life of tilapia fillets stored at 4 ± 1 °C for 10 days. UV samples had the highest reduction of *S. typhimurium* (1.13 log colony forming units/g; CFU/g) and *E. coli* O157:H7 (0.70 log CFU/g). MAP and MAP/UV reduced the growth of *S. typhimurium* in 0.50 log CFU/g and did not affect the growth of *E. coli* O157:H7. UV, MAP, and MAP/UV increased lag phase and/or generation time of all evaluated bacterial groups, decreased pH values, ammonia formation, texture changes, and, in general, the BA formation throughout storage period, and, therefore, UV, MAP, and MAP/UV extended the shelf life for two, three, and at least five days, respectively. MAP/UV, MAP, and UV decreased redness, MAP/UV and MAP increased yellowness and lipid oxidation, while UV did not affect it. MAP/UV demonstrated promising results for shelf life extension; however, different gas ratios in combination with other ultraviolet radiation type C (UV-C) doses should be investigated to reach the highest microbiological safety and maintenance of the overall quality of tilapia fillets.

## 1. Introduction

Nile tilapia (*Oreochromis niloticus*) is one of the main freshwater fish species widely contributing to aquaculture growth and an increase of white fish consumption [1]. Nevertheless, fish is highly perishable due to intense action of autolytic enzymes and high-quality nutrient composition, which serve as a substrate for microbial growth and oxidative reactions resulting in rapid deterioration post-mortem, loss of quality attributes such as color, odor, and texture, and formation of toxic molecules such as ammonia and biogenic amines [2,3]. According to Nowsad et al. [4], 34% of the tilapia fillets lose the quality during transport and in the retail display and, therefore, they are sold at a low price or discarded, resulting in a negative economic impact in the tilapia production chain. 

Besides having a short shelf life, tilapia may be a great vehicle of potential pathogens such as *Salmonella typhimurium* [5] and *Escherichia coli* O157:H7 [6], which represents a public health hazard mainly due to fish that is usually consumed raw or undercooked. According to the Center for Disease Control and Prevention [7], bacteria were the main cause of outbreak-associated illnesses accounting for 37% of the cases in 2017. *Salmonella* spp. and *E. coli* O157:H7 were considered potential foodborne pathogens being responsible for 55% and 9% of the outbreak-associated illnesses, respectively [7]. 

Therefore, to increase distribution, commercialization, and consumption of fish worldwide, the United Nations Food and Agriculture Organization has stimulated studies about effective and viable preservation technologies to reduce the spoilage rate in fresh fish and guarantee food safety [1]. On the other hand, one of the main challenges for the food industry and academia is to reach a maximum shelf life, including the reduction of relevant pathogens while maintaining the original quality attributes of foods [8]. In this way, combined preservation technologies have been widely studied to evaluate the ability of synergistic effects in improving bacterial quality and in minimizing undesirable changes from the use of only one method.

Ultraviolet radiation type C (UV-C) is an effective non-thermal technology against the bacterial growth due to its direct and indirect actions on the microbial DNA through binding between cytosine and thymine and formation of free radicals by water radiolysis, respectively [9,10]. UV-C radiation is an easily implementable technology in the food industry, of low cost, and has a lack in toxic residues [10]. Furthermore, UV-C has been proven effective in reducing pathogens [11,12,13] and in extending the shelf life of refrigerated fish species [14,15]. Nevertheless, UV-C doses needed to extend the shelf life may result in adverse changes in color and texture by reactive oxygen species (ROS)-induced oxidation depending on fish composition and type and load of microorganisms into the fish matrix [3,16]. In this way, UV-C is usually applied in vacuum-packed fish; however, it remains with approximately 5% residual oxygen inside the package mainly due to oxygen penetration through packaging material during the storage period [17]. 

Regarding packaging, some authors have focused on evaluating different packaging materials to improve the shelf life of foods [18,19]. Modified atmosphere packaging (MAP) is a well-known technology for inhibiting the bacterial growth in fish at a low cost, ease implementation, and overall quality maintenance depending on food composition, choice, and ratio of gases used [2,20,21]. MAP replaces the atmosphere air composition inside the package with a gas mixture, mainly carbon dioxide (CO_2_), nitrogen (N_2_), and oxygen (O_2_). CO_2_ is primarily responsible for the bacteriostatic effect due to its dissolution in water and lipids leading to the formation of carbonic acid (H_2_CO_3_) and subsequent acidification of the environment [21]. N_2_ is an inert gas with low solubility in water commonly used to avoid package collapse with high CO_2_ concentration replacing O_2_ in foods in which it can be removed entirely [22]. O_2_ is mainly used to maintain red flesh color and to inhibit the growth of anaerobic bacteria. However, it is unnecessary for tilapia, which has a white color and obligate aerobic bacteria (*Pseudomonas* spp.) as dominant microbiota during refrigerated storage [23,24]. Likewise, the presence of O_2_ could accelerate lipid oxidation due to the high amount of unsaturated fatty acids (66%) in tilapia [3]. Moreover, the absence of O_2_ into the MAP system could prevent the adverse effects induced by ROS from UV-C radiation. 

Despite these facts, the combined effect of MAP and UV-C radiation on quality parameters of tilapia fillets is still unknown. Therefore, the aims of this study were to (1) investigate the effect of combined MAP (50% CO_2_ and 50% N_2_) and UV-C radiation (0.30 J/cm^2^) on the reduction of *Salmonella typhimurium* and *E. coli* O157:H7 in tilapia fillets; and (2) evaluate the effect of these combined preservation technologies on biogenic amines and the shelf life of tilapia fillets stored at 4 ± 1 °C for 10 days. 

## 2. Materials and Methods

### 2.1. Experimental Design

One-hundred and one fresh tilapia (*Oreochromis niloticus*) fillets were purchased from a commercial fish processing facility in Rio de Janeiro, Brazil (22°27′46″ S 042°39′10″ W). Immediately after obtaining, tilapia fillets (100 g each) were transported in polystyrene boxes containing ice to the laboratory within 2 h. Tilapia fillets were randomly divided into five treatments: AP (air-packed tilapia fillets), VP (vacuum-packed tilapia fillets), MAP (MAP-packed tilapia fillets with 50% CO_2_ and 50% N_2_), UV (UV-C treated tilapia fillets at 0.30 J/cm^2^), and MAP/UV (MAP-packed tilapia fillets with 50% CO_2_ and 50% N_2_ treated with UV-C at 0.30 J/cm^2^). 

Fifty fillets underwent an inoculation experiment of which twenty-five were for the experiment with an inoculation of *Salmonella typhimurium* (*n* = 5; 5 treatments × 5 replicates) and twenty-five were for the experiment with an inoculation of *Escherichia coli* O157:H7 (*n* = 5; 5 treatments × 5 replicates). Fifty-one fillets were allocated to a shelf life experiment of which one fillet was immediately analyzed for total aerobic mesophilic count (TAMC), total aerobic psychrotrophic count (TAPC), and *Enterobacteriaceae* count on day 0 and fifty fillets were monitored during the storage period at 4 ± 1 °C (*n* = 10; 5 treatments × 10 replicates).

### 2.2. Packaging Treatments

Tilapia fillets were individually packed into proper nylon/polyethylene bags (80 μm in thickness, 22 cm in height, and 15 cm in width) to vacuum or modified atmosphere packaging with an O_2_ transmission rate (OTR) of 66.31 cc/m^2^/day and water-vapor transmission rate (WVTR) of 4.91 gm/m^2^/day at 23 °C and 50% relative humidity according to the manufacturer’s information (Gabrilina, São Paulo, Brazil). A vacuum-packaging machine equipped with gas injection and thermal sealing devices (AP 450 TECMAQ, São Paulo, Brazil) was used to perform the vacuum (VP and UV) and modified atmosphere (MAP and MAP/UV). The gas mixture used in MAP was 50% CO_2_ and 50% N_2_, and the gases were purchased from Linde (Rio de Janeiro, Brazil). This gas ratio was chosen due to its effectiveness in increasing the shelf life while maintaining the acceptable sensory attributes in tilapia fillets stored under refrigeration [25]. 

### 2.3. UV-C Treatment

A stainless steel barrel-shaped chamber with twelve UV-C lamps (six of 30 W and six of 55 W; OSRAM HNS, OFR, Munich, Germany), developed by Lázaro et al. [26], was used to perform the UV-C radiation. The vacuum-packed (VP) and MAP-packed tilapia fillets (MAP/UV) were individually located in the geometrical center inside of the UV-C equipment with a sample-lamps distance of 14 cm. A UV radiometer (MRUR-203, Instrutherm Ltd.a., São Paulo, Brazil) was wrapped with the same sample packaging and placed next to the sample inside of the UV-C apparatus in each UV-C exposure. It was used to monitor the UV intensity levels every 5 s until reaching a dose of 0.30 J/cm^2^. This UV-C dose was chosen due to its effectiveness in prolonging shelf life with minimal adverse effects in the overall quality of refrigerated tilapia fillets [3,27].

The effect of both MAP and UV-C depends on dose or gas ratios used, food composition, load, and type of microorganisms, which have different adaptation mechanisms [2,3,10,16,20,21]. At the present moment, there are only two studies related to the combined effect of the CO_2_/N_2_ MAP and UV-C. However, they investigated this effect on the reduction of pathogen or shelf life using a combination of a different gas mixture of MAP and UV-C dose, different pathogen, and different fish species [2,20] when compared to the present study. Moreover, these studies did not include protein oxidation, instrumental color, and texture parameters, which are relevant fish freshness indicators. Therefore, the effect regarding the combination of MAP with 50% CO_2_ and 50% N_2_ and UV-C at 0.30 J/cm^2^ on the reduction of *S. typhimurium* and *E. coli* O157:H7, shelf life, biogenic amines, and oxidative parameters is still unknown in any fish species. 

### 2.4. Inoculation Experiment 

#### 2.4.1. Preparation of Inoculum and Application into Tilapia Fillets

Serotypes of *Salmonella typhimurium* (ATCC 14028) and *Escherichia coli* O157:H7 CDC EDL-933 (ATCC 43895) obtained from the Collection of Reference Microorganisms, National Institute of Control Quality in Health (FIOCRUZ, Rio de Janeiro, Brazil), were prepared according to Lázaro et al. [26]. In brief, each serotype was twice suspended in brain heart infusion broth (BHI; Himedia, India) at 37 °C for 24 h, cultures were centrifuged at 1000× *g* at 4 °C for 15 min, and then the sediment was washed three times with phosphate-buffered saline (PBS; Himedia, India) at pH 7.2–7.4. The bacterial concentrations (1 mL = approximately 4.0 × 10^8^ cells) were determined by UV spectrophotometer (Smartspec Plus, Bio-Rad, Hercules, CA, USA) at 600 nm.

The bacterial inoculum (1 mL) was spotted on the surface of each tilapia fillet (two sides) using a micropipette, and the inoculum was massaged onto the fillets following a rest period of 15 min at 20 °C to ensure complete absorption. Then, tilapia fillets were packed in vacuum (VP and UV) or MAP (MAP and MAP/UV), subsequently subjected to UV-radiation (UV and MAP/UV), and stored at 4 ± 1 °C. After 24 h, all treatments were analyzed in duplicate for *S. typhimurium* and *E. coli* O157:H7. 

#### 2.4.2. Bacteriological Analyses

Samples were obtained by a wet swab technique as described by Røssvoll et al. [28] with modifications. The swab was rubbed vertically and horizontally ten times across the sampling site (10 cm^2^), delineated by a template. After this procedure, the swab was homogenized in 100 mL of peptone water (0.1%). Suspensions were plated on Salmonella Shigella agar (Himedia, Mumbai, India) and Fluorocult Violet Red Bile agar (VRB-agar; Merk, Darmstadt, Germany) through a Spiral Plater (Eddy Jet 2, IUL Instruments, Barcelona, CAT, Spain) for *Salmonella typhimurium* and *Escherichia coli* O157:H7 counts, respectively. Then, plates were incubated at 37 °C for 48 h, and after this period, colonies were counted using an electronic counter (Flash & Go, IUL instruments, Leerdam, The Netherlands). The results were expressed as log CFU (colony forming units)/g [29]. 

### 2.5. Shelf Life Experiment 

After tilapia fillets were packed in vacuum (VP and UV) or MAP (MAP and MAP/UV) and immediately subjected to UV-radiation (UV and MAP/UV), fillets were stored at 4 ± 1 °C for 10 days and analyzed daily for TAMC, TAPC, and *Enterobacteriaceae* count, biogenic amines, pH, ammonia, lipid oxidation, instrumental color parameters, and texture profile. TAMC, TAPC, *Enterobacteriaceae* count, biogenic amines, ammonia, and lipid oxidation were performed in duplicate, pH in triplicate, instrumental color parameters in sextuplicate, and texture profile in quadruplicate. 

#### 2.5.1. Bacteriological Analyses 

All analyses were conducted following the standard microbiological methods [29]. For TAMC and TAPC, dilutions were plated on plate-count agar (PCA, Merck, Darmstadt, Germany), and for *Enterobacteriaceae* count on Violet Red Bile Glucose agar (VRBG-agar, Merck, Darmstadt, Germany). Plates for TAMC, TAPC, and *Enterobacteriaceae* count were incubated at 37 °C for 48 h, 10 °C for 7 days, and 35 °C for 24 h, respectively. Furthermore, plating and counting of colonies were performed in duplicate with Spiral Plater (Eddy Jet 2, IUL Instruments, Barcelona, Spain) and electronic counter (Flash & Go, IUL instruments, Leerdam, UT, The Netherlands). The results were expressed as log CFU/g.

#### 2.5.2. Quantification of Biogenic Amines 

Histamine, tyramine, cadaverine, putrescine, spermidine, and spermine were determined as the method of Lázaro et al. [30] through High-Performance Liquid Chromatography (HPLC). The chromatographic system consisted of a Shimadzu Prominence UFLC apparatus (Shimadzu, Kyoto, Japan) equipped with a DGU-20A5 degasser, a SIL-20AC autosampler, an LC-20AD quaternary pump, a CTO-20A column oven, an SPD-M20A diode array detector, and a CBM-20A communication bus module. The separation of the biogenic amines (BAs) was performed on a C18 Spherisorb ODS2 (15 × 0.46 cm id., 5 μm, Waters) column equipped with a Supelco Ascentis C18 (2 × 0.40 cm id., 5 μm) guard column under isocratic conditions. The mobile phase consisted of 42:58 (*v*/*v*) of acetonitrile (Tedia^®^) and ultrapure water (Simplicity-Millipore, Molsheim, France). The chromatography conditions were as follows: flow rate of 1 mL/min, injection volume of 20 µL, and column temperature of 20 °C. BAs were detected by UV absorption at 198 nm after a total run time of 15 min. Between each sample, a 10 min cleaning step was performed with acetonitrile. The biogenic amines were identified by retention time, quantified by peak area using external standards, and the results were expressed in mg/kg. This analysis was performed in duplicate.

#### 2.5.3. Determination of pH and Ammonia Levels

The pH values were measured as the method described by Conte-Junior et al. [21]. A sample aliquot of 10 g was homogenized with 90 mL of distilled water, and the pH was measured with a digital pH meter (Digimed^®^ DM-22) equipped with a DME-R12 electrode (Digimed^®^). This analysis was performed in triplicate. 

The quantification of ammonia was performed according to the colorimetric method of McCullough [31] modified by Rodrigues et al. [2] by using a UV-1800 spectrophotometer (Shimadzu, Kyoto, Japan). Results were expressed in μg ammonia (NH_3_)/g. This analysis was performed in duplicate.

#### 2.5.4. Measurement of Lipid Oxidation

Lipid oxidation was determined using the distillation method of 2-thiobarbituric acid reactive substances (TBARS), according to Tarladgis et al. [32] and modified by Monteiro et al. [33]. The absorbance values were measured at 528 nm on a Smartspec Plus spectrophotometer (Bio-Rad, Hercules, CA, USA), and the results were expressed as mg malondialdehyde (MDA)/kg. This analysis was carried out in duplicate.

#### 2.5.5. Determination of Instrumental Color Parameters

After a blooming period of 30 min at 20 °C, lightness (L*), redness (a*), and yellowness (b*) values were measured at six random locations on the surface of each fillet through a Minolta CM-600D portable spectrophotometer (Konica Minolta Sensing, Inc., Osaka, Japan) using illuminant A, 10° standard observer, and 8 mm-diameter aperture [34]. Additionally, the total color difference (ΔE) between days 10 and 0 of the storage under refrigeration was calculated for each treatment based on the equation proposed by AMSA [34]:

ΔE_10-0_ = [(L^*^ − L_0_^*^)^2^ + (a^*^ − a_0_^*^)^2^ + (b^*^ − b_0_^*^)^2^]^1/2^.

#### 2.5.6. Determination of Texture Profile 

The instrumental texture parameters (hardness, chewiness, cohesiveness, springiness, and resilience) were measured in four pieces (2 × 2 × 2 cm^3^) from each fillet through TA.XTplus Texture Analyser (Stable Micro Systems, Surrey, UK) coupled to a cylindrical P/36 R probe following the conditions described by Sun et al. [35]. 

### 2.6. Statistical Analyses

The bacterial growth curves and bacterial growth parameters (lag phase—lag, generation time—GT, and number of colonies in the stationary phase—NC) were obtained through a DMFit predictive microbiology software (available at http://www.combase.cc) using the primary predictive model [36]. The total amount of each physicochemical parameter produced during the 10 days of refrigerated storage was calculated by area under the curve (AUC) using GraphPad Prism 5 (Graphpad Software Inc., San Diego, CA, USA) at a 5% of confidence level. The differences for AUC among treatments were detected by one-way ANOVA with Tukey’s post-hoc test (*p* < 0.05). One-way ANOVA with Tukey’s post-hoc test (*p* < 0.05) was also used to identify differences among treatments concerning *Salmonella typhimurium* and *E. coli* O157:H7 counts, bacterial growth parameters of TAMC, TAPC, and *Enterobacteriaceae*, and total color difference (∆E). Two-way ANOVA with Tukey’s post-hoc test (*p* < 0.05) was used to identify differences between treatments (AP, VP, UV, MAP, and MAP/UV) and days of storage (1, 2, 3, 4, 5, 6, 7, 8, 9, and 10) for all evaluated physicochemical parameters. All analysis of variance and Tukey’s tests were performed using the XLSTAT software, version 2012.6.08 (Addinsoft, New York, NY, USA).

## 3. Results and Discussion

### 3.1. Effect of MAP and UV-C on Reduction of Pathogenic Bacteria 

VP did not affect the growth of *S. typhimurium* and *E. coli* O157:H7 (*p* > 0.05; Table 1). MAP and MAP/UV were equally effective for *S. typhimurium* reducing its growth in 0.50 log CFU/g. However, both MAP and MAP/UV were ineffective against *E. coli* O157:H7 (*p* > 0.05). UV was the most effective method for the reduction of *S. typhimurium* and *E. coli* O157:H7, decreasing it by 1.13 and 0.70 log CFU/g, respectively (*p* < 0.05; Table 1). 

Our results may be attributed to an antimicrobial effect of MAP and UV-C [9,10,21,37] considering that mechanisms of the CO_2_ are further complex involving direct action in one or more bacterial mechanisms such as changes in uptake and absorption of nutrients, enzyme reactions, intracellular pH, and physicochemical properties by CO_2_ penetration into bacterial membranes [38]. 

MAP/UV was ineffective against the growth of both evaluated pathogens, and it was better observed for *Escherichia coli* O157:H7. These findings may be explained because MAP can impair the penetration power of the UV-C radiation at 0.30 J/cm^2^ as well as to the higher ability of adaptation of *E. coli* compared to *S. enterica* [20,39,40]. Rodrigues et al. [20] observed that MAP (50% CO_2_/50% N_2_) was a barrier to penetration of UV-C radiation in rainbow trout fillets. However, it was found for doses (0.04, 0.06, and 0.10 J/cm^2^) at least three times less than the dose used in this study. Furthermore, although there are similar genetic and metabolic characteristics between the two evaluated bacterial species, each species presents different genetic pathways to adaptation and multiplication in identical growth conditions [39,40]. Knöppel et al. [40] evaluated the adaptation of *Salmonella enterica* and *E. coli* in complex media such as foods and under stress conditions and observed that both bacteria express adaptation mechanisms in lag or stationary phases; however, it was higher in *E. coli* than in *S. enterica*. 

In agreement with our results, MAP with 50% CO_2_ reduced the growth of Salmonella multi-strains in 0.8 log CFU/g in minced meat [41]. On the other hand, MAP with 30% CO_2_/69.6% N_2_/0.4% CO and 30% CO_2_/70% O_2_ did not affect the reduction of *E. coli* in refrigerated lamb meat stored [42]. UV-C at 0.06 and 0.23 J/cm^2^ reduced the growth of Salmonella multi-strains in 0.34 and 0.57 log CFU/g, respectively, in chicken meat [26]. Sommers et al. [13] observed a reduction of 0.60 log CFU/g for *E. coli* in chicken breast meat treated with UV-C at 0.011 to 0.013 J/cm^2^. There are no studies about combined MAP (50% CO_2_/50% N_2_) and UV-C radiation (0.30 J/cm^2^) on the reduction of *Salmonella* spp. and *E. coli* in meat. However, Rodrigues et al. [20] reported that combined MAP (30% CO_2_/70% N_2,_ 50% CO_2_/50% N_2_, and 70% CO_2_/30% N_2_) and UV-C radiation (0.04, 0.06, and 0.10 J/cm^2^) were ineffective on the reduction of *Proteus mirabilis* in rainbow trout fillets. It is worth highlighting that *Proteus mirabilis* is a Gram-negative and facultative anaerobic bacteria, as well as *S. typhimurium* and *E. coli* O157:H7 [23]. 

### 3.2. Effect of MAP and UV-C on Biogenic Amines and Overall Quality Parameters of Tilapia Fillets During Refrigerated Storage

#### 3.2.1. Bacterial Growth

TAMC, TAPC, and *Enterobacteriaceae* count increased throughout the storage period in all treatments (*p* < 0.05; Supplementary File S1; Table 2). 

According to the International Commission on Microbiological Specifications for Foods [43], refrigerated fish is unfit for consumption when the aerobic plate count achieves 7 log CFU/g. As *Pseudomonas* spp. is the main spoilage bacteria in fish stored aerobically at low temperatures [23], the shelf life of tilapia fillets was established based on TAPC. In our study, AP, VP, MAP, and UV exceeded the limit of 7.0 log CFU/g for TAPC on days 5, 6, 8, and 7, respectively, while MAP/UV did not reach this limit throughout the entire storage period (Supplementary File S1). These findings may be attributed to different effects of the preservation treatments on the bacterial growth parameters. 

For TAPC, VP had a higher GT than AP (*p* < 0.05; Table 2), which may be explained by the high sensitivity of *Pseudomonas* spp., which are obligate aerobic bacteria, to low O_2_ concentrations [2,44]. UV exhibited higher GT (*p* < 0.05) compared to VP, probably due to the antimicrobial effect of the UV-C radiation [10]. MAP and AP had a similar GT (*p* > 0.05), and MAP showed a lower GT (*p* < 0.05) than VP and UV; however, MAP was the only treatment with lag (Table 2). This phenomenon may be attributed to sublethal injury on aerobic psychrotrophic bacteria caused by 50% CO_2_, which grew more slowly at the beginning of the storage period (lag phase formation), and more rapidly after the adaptation period mainly due to an environment with low bacterial competition for nutrients [45], resulting in a shorter GT. Therefore, our results indicate that the adaptation period of aerobic psychrotrophic bacteria to 50%CO_2_/50%N_2_ mixture was determinant to a higher shelf life extension in MAP-packed tilapia fillets in comparison with the fillets submitted to isolated treatments (AP, VP, and UV). MAP/UV showed the highest GT (*p* < 0.05), probably due to the synergistic effect of both MAP and UV-C against bacterial growth [2]. 

The results of the *Enterobacteriaceae* count were similar to TAPC, except the GT for AP and VP (Table 2). In addition to Gram-negative aerobic bacteria (e.g., *Pseudomonas* spp.), the microbiota of tilapia also contains an abundance of Gram-negative facultative anaerobic bacteria from the *Enterobacteriaceae* family [23,46]. Gram-negative bacteria do not have a thick outer cell membrane, which serves as a barrier to protect the microbial DNA, and, therefore, they are highly sensitive to UV-C radiation and CO_2_ [47,48]. These facts explain our results of MAP, UV, and MAP/UV compared to AP, VP, and isolated MAP and UV treatments, respectively. Concerning AP and VP, both treatments had a similar GT (*p* > 0.05; Table 2), which may be explained because the *Enterobacteriaceae* family is composed of facultative anaerobic bacteria, which are capable of growing at low or the absence of oxygen [23]. 

Regarding TAMC, AP and VP showed similar lag and GT (*p* > 0.05), while MAP demonstrated a higher lag and GT than AP (*p* < 0.05; Table 2). MAP and VP had similar lag (*p* > 0.05); however, MAP exhibited a higher GT than VP (*p* < 0.05). UV had a similar GT to MAP and MAP/UV (*p* > 0.05), while MAP/UV showed the highest lag and a higher GT than AP, VP, and MAP (*p* < 0.05; Table 2). These results can be explained by two main reasons. First, mesophilic bacteria need an adaptation period to grow at a refrigeration temperature of up to 15 °C [23]. Second, a predominance of obligate aerobic bacteria, facultative anaerobic bacteria, and other Gram-negative bacteria with high sensitivity to UV-C and CO_2_ such as *Shewanella putrefaciens*, *Aeromonas* spp., and *Chromobacterium violaceum* are also natural in the microbiota of tilapia [23,46]. It is worth highlighting that all treatments demonstrated lag, except UV (Table 2). This fact may be due to UV-C acting only on the surface of the foods, and it may cause protein degradation, which increases the bioavailability of nutrients on the environment, allowing a rapid bacterial adaptation [10]. On the other hand, the highest lag was observed for MAP/UV. Therefore, the present study suggests that the generation of ROS from water radiolysis caused by UV-C radiation may be intensified by the release of water from H_2_CO_3_ formation in CO_2_ MAP. It may have induced a higher sublethal injury on the mesophilic bacterial group leading to a longer lag when compared to MAP applied alone. 

In agreement with our results, authors have reported the effectiveness of UV-C radiation and CO_2_/N_2_ MAP on shelf life extension of fish species stored under refrigeration [14,15,24,49]. Regarding combined preservation methods, there is only one study reporting that the shelf life of refrigerated rainbow trout fillets subjected to a high CO_2_ MAP (80% CO_2_/20% N_2_) and a low UV-C dose (0.10 J/cm^2^) was at least twice bigger than air-packed trout fillets [2].

#### 3.2.2. Biogenic Amines 

Overall, although some fluctuations have been observed during the storage period, biogenic amines (histamine, tyramine, cadaverine, and putrescine) production increased during refrigerated storage (*p* < 0.05), except the tyramine production in UV and MAP/UV, which remained constant (*p* > 0.05; Supplementary File S2). In general, the production of spermidine and spermine in AP increased only in the end of the storage period, while it was constant throughout the refrigerated storage period in the other treatments (VP, MAP, UV, and MAP/UV). Moreover, histamine, tyramine, and cadaverine decreased after day 8 (*p* < 0.05). The production of biogenic amines is a multifactorial process dependent on several factors such as amount of precursor amino acids (substrate), bacterial growth kinetics, as well as its proteolytic action and decarboxylase activity [50]. Therefore, the decrease in some biogenic amines may be associated with a low amount of substrate and/or low activity of microorganisms. 

**Figure 1:**
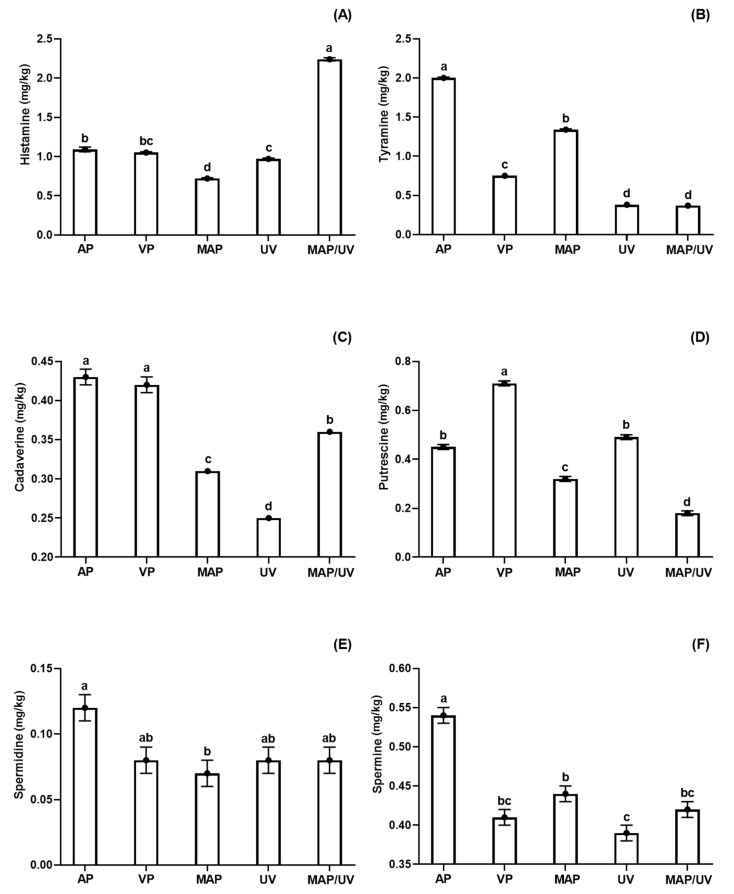
Total histamine (**A**), tyramine (**B**), cadaverine (**C**), putrescine (**D**), spermidine (**E**), and spermine (**F**) in tilapia (*Oreochromis niloticus*) fillets non- and treated with ultraviolet radiation (UV-C) and modified atmosphere packaging (MAP) throughout the entire storage period (10 days) at 4 ± 1 °C. ^a,b,c,d^ Different letters indicate significant differences (*p* < 0.05) among treatments. AP—air-packed tilapia fillets, VP—vacuum-packed tilapia fillets, MAP—MAP-packed tilapia fillets with 50% CO_2_ and 50% N_2_, UV—UV-C treated tilapia fillets at 0.30 J/cm^2^, and MAP/UV—MAP-packed tilapia fillets with 50% CO_2_ and 50% N_2_ treated with UV-C at 0.30 J/cm^2^.

Concerning histamine, MAP/UV had the highest production, followed by MAP and UV, during the entire storage period (*p* < 0.05; Figure 1A). No difference was observed between VP and UV, and between AP and VP (*p* > 0.05; Figure 1A). Therefore, isolated MAP and UV-C were effective in decreasing the formation of histamine, while MAP/UV increased the formation of this amine throughout refrigerated storage compared to AP. Regarding tyramine, AP showed the highest production followed by MAP, VP, UV, and MAP/UV (*p* < 0.05), which did not differ from each other over the storage period (*p* > 0.05; Figure 1B). With regards to cadaverine, a similar production was found for AP and VP (*p* > 0.05); however, the other treatments (MAP, UV, and MAP/UV) exhibited lower cadaverine production than AP and VP throughout the refrigerated storage period (*p* < 0.05; Figure 1C). UV had the lowest cadaverine production followed by MAP and MAP/UV (*p* < 0.05; Figure 1C). Regarding putrescine, MAP/UV demonstrated the lowest production followed by MAP, UV, and AP (*p* < 0.05), which did not differ from each other during the entire storage period (*p* > 0.05; Figure 1D). VP exhibited the highest putrescine production throughout the storage period (*p* < 0.05; Figure 1D). Therefore, when compared to AP, UV did not affect the formation of putrescine, while MAP was able to decrease the formation of this amine, and MAP/UV was the most effective method to decrease the formation of putrescine during the refrigerated storage period. Regarding spermidine, the only difference observed was a lower production in MAP compared to AP throughout the entire storage period (*p* < 0.05; Figure 1E). Despite this fact, VP, MAP, UV, and MAP/UV showed a lower numeric value for spermidine than AP throughout the refrigerated period (Figure 1E). Concerning spermine, AP exhibited the highest production throughout the storage period (*p* < 0.05; Figure 1F). UV showed lower spermine production than MAP (*p* < 0.05), and no difference was found in the spermine production among UV, VP, and MAP/UV, and among MAP, VP, and MAP/UV (*p* > 0.05; Figure 1F).

The increase of biogenic amines naturally occurs during refrigerated storage, and it is usually associated with the action of amino acid decarboxylase bacteria mainly from the *Enterobacteriaceae* family [50,51]. Due to the antimicrobial effect of UV-C and CO_2_ MAP, it is expected that both preservation methods decrease the production of biogenic amines in fish stored under refrigeration. Nevertheless, the production of biogenic amines may vary depending on microbiota (type and initial load) and amino acid composition, including the amount available of precursor amino acids [50]. Furthermore, bacterial groups belonging to the same family may have different sensibilities to a preservation treatment, and the preservation methods may change the food components depending on food matrix composition and processing conditions such as dose or gas ratio used [2,3,16,21,23,37]. 

Both MAP (50% CO_2_/50% N_2_) and UV-C radiation (0.30 J/cm^2^), alone or in combination, were effective against the growth of *Enterobacteriaceae.* However, unexpectedly, MAP/UV increased the formation of histamine, and UV-C did not affect the formation of putrescine over the storage period. These results may be explained because CO_2_ can increase the availability of amino acids by protein denaturation [52], and UV-C radiation may catalyze the production of Fe^3+^, leading to oxidative decarboxylation of amino acids [53,54]. Moreover, lactic acid bacteria may also produce biogenic amines in fish [55]. This bacterial group grows in microaerobic or anaerobic conditions and are Gram-positive bacteria, which are more resistant to CO_2_ and UV-C radiation due to the presence of a thick outer cell membrane [23,47,56,57].

The literature is variable and inconsistent about the formation of biogenic amines in fish treated with preservation methods under refrigerated storage, probably due to the high number of influencing factors on their production. Yew et al. [58] reported that MAP with 60% CO_2_ decreased the formation of histamine, putrescine, cadaverine, tyramine, spermidine, and spermine in refrigerated Indian mackerel. On the contrary, these same authors observed that MAP with 30% CO_2_ increased the formation of tyramine and spermidine, but did not affect spermine levels over the storage period. Santos et al. [59] demonstrated that UV-C at 0.10 J/cm^2^ retarded the formation of histamine, cadaverine, putrescine, spermidine, and spermine in pirarucu fillets stored at 4 °C. Monteiro et al. [27] observed no effect of UV-C radiation (0.10 and 0.30 J/cm^2^) on the production of cadaverine, putrescine, and spermidine in tilapia fillets stored under refrigeration. Rodrigues et al. [2] reported that combined MAP (80% CO_2_/20% N_2_) and UV-C (0.10 J/cm^2^) were the most effective methods in decreasing cadaverine production. However, it did not affect putrescine production throughout the storage period during refrigerated storage of rainbow trout. 

Concerning regulatory limits, histamine is the only biogenic amine with legal limits. According to European Comission [60], scombroid fish and fish products must contain the maximum amount of 200 and 400 mg of histamine/kg of fish muscle, respectively. The Food and Drug Administration [61] establishes 500 mg of histamine/kg of fish muscle as a legal limit. In comparison, the Brazilian legislation [62] determines a maximum amount of 100 mg of histamine/kg of fish muscle for scombroid fish. In the present study, all treatments showed histamine levels very much below the national and international limits throughout the storage period. 

#### 3.2.3. Fish pH

**Figure 2:**
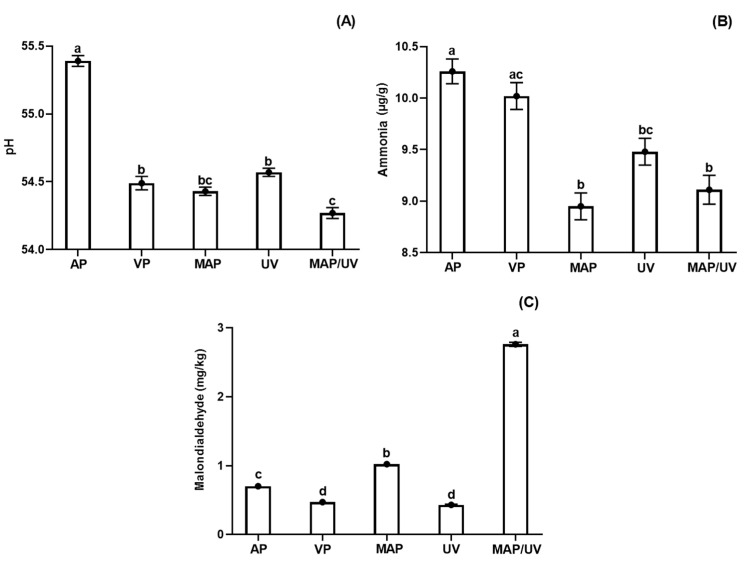
Total pH (**A**), ammonia (**B**), and malondialdehyde (**C**) in tilapia (*Oreochromis niloticus*) fillets non- and treated with ultraviolet radiation (UV-C) and modified atmosphere packaging (MAP) throughout the entire storage period (10 days) at 4 ± 1 °C. ^a,b,c,d^ Different letters indicate significant differences (*p* < 0.05) among treatments. AP—air-packed tilapia fillets, VP—vacuum-packed tilapia fillets, MAP—MAP-packed tilapia fillets with 50% CO_2_ and 50% N_2_, UV—UV-C treated tilapia fillets at 0.30 J/cm^2^, and MAP/UV—MAP-packed tilapia fillets with 50% CO_2_ and 50% N_2_ treated with UV-C at 0.30 J/cm^2^.

Although fluctuations on pH values of all treatments during the entire refrigerated storage period, untreated and all treated tilapia fillets showed lower pH values on day 10 compared to day 1 (*p* < 0.05; Supplementary File S3). AP had the lowest reduction of pH during the storage period (*p* < 0.05; Figure 2A). No difference was found for pH values among VP, MAP, and UV, and between MAP and MAP/UV (*p* > 0.05); however, MAP/UV exhibited a higher reduction of pH than VP and UV throughout the storage period (*p* < 0.05; Figure 2A). These findings indicate that UV-C did not affect the pH values and, in general, MAP was responsible for the highest reduction of pH values throughout the refrigerated storage period. 

The decrease of pH during refrigerated storage may be attributed to a formation imbalance of alkaline and acid compounds from the action of endogenous and microbial enzymes during post-mortem degradation associated with buffering capacity, which is related to specific biochemistry characteristic of each muscle [15,63,64]. The results of pH values observed for VP, MAP, UV, and MAP/UV may be explained by the mechanism of action of CO_2_ and by the growth of lactic acid bacteria, which can grow in microaerobic or anaerobic environments and are Gram-positive, being more resistant to CO_2_ and UV-C radiation [21,23,37,47,56]. 

Similar findings were previously reported with the application of MAP and UV-C radiation, alone or in combination, in fish species [2,14,65]. However, Rodrigues et al. [2] did not observe a higher reduction of pH values comparing 80% CO_2_/20% N_2_ MAP and vacuum packaging in rainbow trout fillets stored at 4 °C for 22 days.

#### 3.2.4. Ammonia

Although variations (increase and decrease) found for ammonia values during the storage period in all treatments, untreated and all treated tilapia fillets exhibited an increase of ammonia values throughout refrigerated storage (*p* < 0.05; Supplementary File S3). It was expected because ammonia is an alkaline compound formed from protein degradation by the action of endogenous proteases and spoilage microorganisms, which naturally occur during the post mortem period in fish [66]. Regarding fluctuations in ammonia values during storage, it may be attributed to the binding capacity of the MDA with ammonium compounds from protein degradation [67]. 

VP had similar production of ammonia to AP during the entire storage period (*p* > 0.05). However, lower production of ammonia was observed for MAP, UV, and MAP/UV when compared to AP and VP (*p* < 0.05), except UV, which did not differ from VP (*p* > 0.05; Figure 2B). Moreover, despite no difference found in the production of ammonia among MAP, UV, and MAP/UV (*p* > 0.05), UV showed a higher numeric value for ammonia than MAP and MAP/UV throughout the refrigerated period. These results may be explained by our results of TAPC together with the protein changes caused by UV-C radiation and CO_2_ MAP systems, increasing the amount of free amino acids, which are the main substrate for the formation of ammonia [16,27,52].

In agreement with our findings, previous studies reported similar results for ammonia in freshwater fish species treated with isolated or combined MAP and UV-C radiation and stored under refrigeration [2,15,27,33].

#### 3.2.5. Lipid Oxidation

MDA values also fluctuated in all treatments during the storage period as well as the results of ammonia (*p* < 0.05; Supplementary File S3), which may be explained by binding between MDA and ammonium compounds [67]. Despite this fact, all treatments showed an increase in MDA values throughout refrigerated storage (*p* < 0.05; Supplementary File S3). The increase of lipid oxidation in fish stored under refrigeration is well known [2,15,16], and it is usually related to the action of endogenous lipoxygenases and pro-oxidant agents such as oxygen and free iron [68,69].

During the entire storage period, MAP/UV showed the highest formation of MDA followed by MAP, AP, VP, and UV (*p* < 0.05), which did not differ from each other (*p* > 0.05; Figure 2C). The lower MDA values for VP and UV compared to other treatments can be attributed to a very low O_2_ level in the vacuum packaging and no effect of UV-C radiation on lipid oxidation due to a low lipid content in raw tilapia fillets (1.7%) [15,17,70]. The results of MDA values for MAP can be explained by the fact that CO_2_ might denature proteins releasing free iron, which accelerates the lipid oxidation [52]. The findings for the combined treatment (MAP/UV) were unexpected. We hypothesize that the water released from H_2_CO_3_ formation in CO_2_ MAP systems may have helped the water radiolysis via UV-C radiation accelerating generation of ROS and leading to the highest formation of MDA throughout refrigerated storage. 

Corroborating with our results, Monteiro et al. [15], Olatunde et al. [65], and Rodrigues et al. [2] also observed similar findings for lipid oxidation in refrigerated fish treated with UV-C and MAP alone. In disagreement with our results, Rodrigues et al. [2] reported no difference in lipid oxidation between MAP and MAP + UV-C in trout fillets stored at 4 °C. It may be due to differences between studies related to UV-C dose, CO_2_ level, amount, and composition of lipids of the evaluated fish species. 

#### 3.2.6. Instrumental Color Parameters

**Figure 3:**
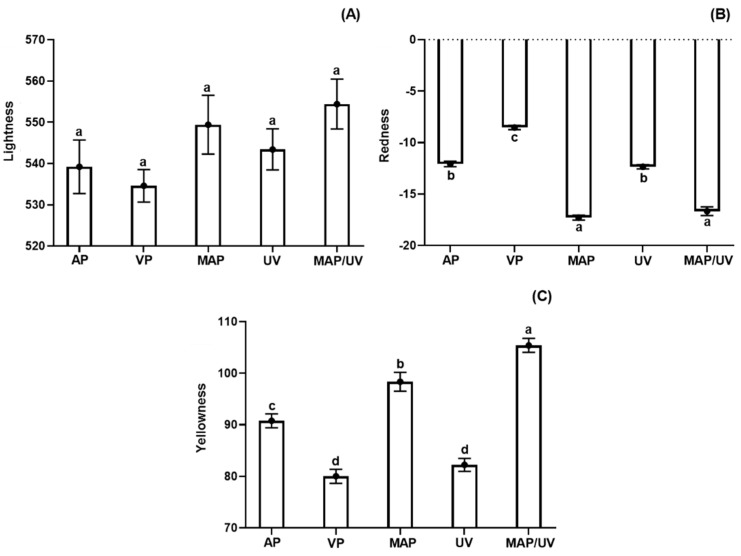
Total lightness (L*; **A**), redness (a^*^; **B**), and yellowness (b^*^; **C**) in tilapia (*Oreochromis niloticus*) fillets non- and treated with ultraviolet radiation (UV-C) and modified atmosphere packaging (MAP) throughout the entire storage period (10 days) at 4 ± 1 °C. ^a,b,c,d^ Different letters indicate significant differences (*p* < 0.05) among treatments. AP—air-packed tilapia fillets, VP—vacuum-packed tilapia fillets, MAP—MAP-packed tilapia fillets with 50% CO_2_ and 50% N_2_, UV—UV-C treated tilapia fillets at 0.30 J/cm^2^, and MAP/UV—MAP-packed tilapia fillets with 50% CO_2_ and 50% N_2_ treated with UV-C at 0.30 J/cm^2^.

Although fluctuations (increase and decrease) observed for L* values throughout the refrigerated storage, no difference was observed between days 10 and 1 for all treatments indicating no changes in L* values during the storage period (*p* > 0.05; Supplementary File S4). Likewise, all treatments exhibited similar L* values during the storage period (*p* > 0.05; Figure 3A). Similarly, Hernández et al. [71] and Monteiro et al. [27] reported no effect of MAP (60%CO_2_/40%N_2_ and 70%CO_2_/30%N_2_) and UV-C radiation (0.10 and 0.30 J/cm^2^) on L* values of sous vide of pirarucu and tilapia fillets stored under refrigeration, respectively. 

All treatments showed a decreasing trend of a* values throughout refrigerated storage, exhibiting lower a* values on day 10 compared to day 1 (*p* > 0.05; Supplementary File S4). During the storage period, MAP and MAP/UV showed the highest reduction of a* values followed by AP, UV, and VP (*p* < 0.05; Figure 3B). No difference was observed in a* values between MAP and MAP/UV, and between AP and UV (*p* > 0.05; Figure 3B). On the other hand, all treatments showed an overall increasing tendency for b* values during the entire storage period. However, it was more marked in the MAP/UV (*p* < 0.05; Supplementary File S4). Throughout the refrigerated storage period, MAP/UV showed the highest b* values followed by MAP, AP, VP, and UV (*p* < 0.05), which did not differ from each other (*p* > 0.05; Figure 3C). 

The decrease in a* values is commonly used as an indicator of meat discoloration. It occurs due to metmyoglobin (MetMb) accumulation from myoglobin autoxidation characterized by the conversion of Fe^2+^ (ferrous oxymyoglobin) to Fe^3+^ (ferric metmyoglobin–MetMb) [72,73]. The results of VP may be due to very low oxygen levels in the vacuum packaging [17]. UV increased the discoloration throughout the storage period, which can be explained by its pro-oxidant effect [53,74]. The results of MAP and MAP/UV may be attributed to protein denaturation induced by CO_2_, making myoglobin more susceptible to autoxidation [52,72]. Furthermore, the reason why there is no difference in the a* values between MAP and MAP/UV could be explained by the capacity of UV-C to oxidize thiol groups with subsequent formation of new disulfide bonds [75]. We hypothesize that newly formed disulfide bonds are more stable to denaturation promoted by CO_2_.

The increase in b* values may be associated with an increase of lipid oxidation during refrigerated storage of fish species [27,65], corroborating with and explaining our results for all treatments. 

Similar to our findings, Merlo et al. [76] found that MAP (100% CO_2_) decreased a* values in salmon fillets under refrigerated storage. Olatunde et al. [65] observed an increase in the b* values in refrigerated Asian sea bass slices packed with MAP (80% CO_2_ and 60% CO_2_). Regarding UV-C radiation, Monteiro et al. [3] reported that 0.10 J/cm^2^ caused discoloration by the increase of protein oxidation. However, it did not affect lipid oxidation and b* values in tilapia fillets stored at 4 °C. This could be explained by the fact that ROS from water radiolysis caused by UV-C affects proteins more strongly than lipids [77].

The total color difference (ΔE_10-0_) was influenced by the treatments (*p* < 0.05; Supplementary File S4). MAP/UV and MAP exhibited the highest ΔE values, followed by AP, VP, and UV (*p* < 0.05; Supplementary File S4). No difference was observed in the ΔE values between MAP/UV and MAP, and between VP and UV (*p* > 0.05; Supplementary File S4). According to Francis [78], ΔE values above 5 means visually perceptible color changes to the human eye, while ΔE values above 12 may be visually perceptible to untrained panelists. Therefore, although color changes in AP, MAP, and MAP/UV are noticeable by the human eye, consumers could not perceive the color changes in any of the treatments.

#### 3.2.7. Instrumental Texture Profile

**Figure 4:**
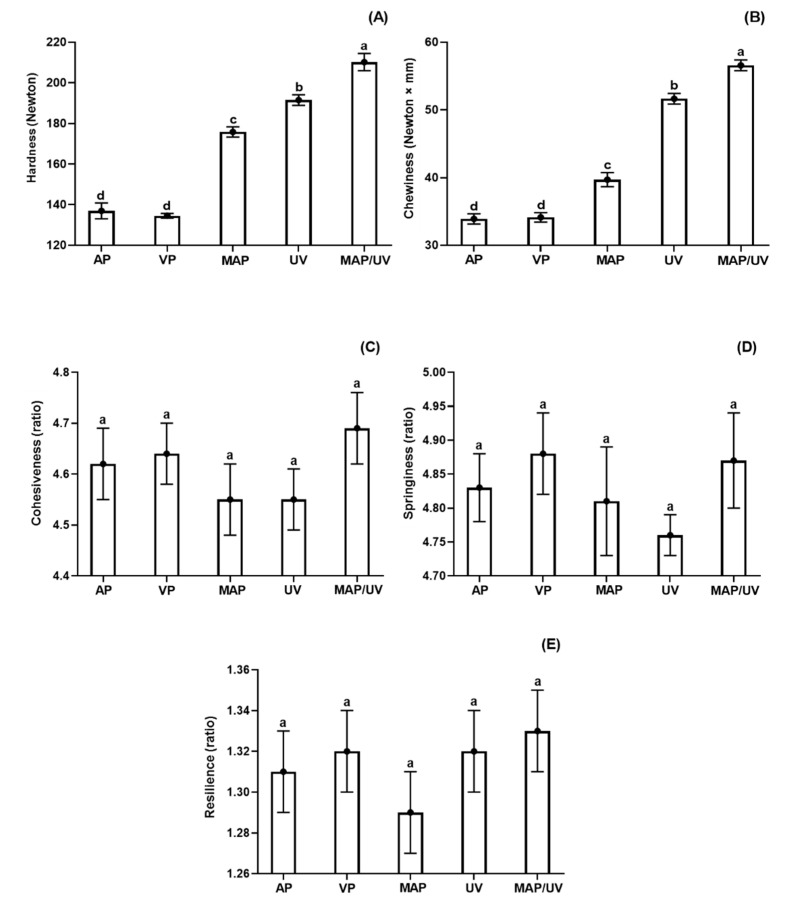
Total hardness (**A**), chewiness (**B**), cohesiveness (**C**), springiness (**D**), and resilience (**E**) in tilapia (*Oreochromis niloticus*) fillets non- and treated with ultraviolet radiation (UV-C) and modified atmosphere packaging (MAP) throughout the entire storage period (10 days) at 4 ± 1 °C. ^a,b,c,d^ Different letters indicate significant differences (*p* < 0.05) among treatments. AP—air-packed tilapia fillets, VP—vacuum-packed tilapia fillets, MAP—MAP-packed tilapia fillets with 50% CO_2_ and 50% N_2_, UV—UV-C treated tilapia fillets at 0.30 J/cm^2^, and MAP/UV—MAP-packed tilapia fillets with 50% CO_2_ and 50% N_2_ treated with UV-C at 0.30 J/cm^2^.

The hardness and chewiness decreased (*p* < 0.05), while cohesiveness, springiness, and resilience remained stable during the entire storage period in all treatments (*p* > 0.05; Supplementary File S5). MAP/UV showed the highest hardness and chewiness followed by UV, MAP, VP, and AP (*p* < 0.05), which had similar hardness and chewiness throughout the refrigerated storage period (*p* > 0.05; Figure 4A,B). No difference was observed for cohesiveness, springiness, and resilience among all treatments during the storage period (*p* > 0.05; Figure 4C–E). 

It is well known that hardness and chewiness decrease in fish species during refrigerated storage [27,79,80]. This phenomenon is mainly related to proteolysis by the action of endogenous and microbial proteases [79]. As denaturation makes the protein more prone to enzymatic actions, our results of hardness and chewiness may be explained by our findings of bacterial growth in association with a* and ammonia values.

In agreement with our results, Xie et al. [81] reported that MAP (60% CO_2_) was effective in maintaining hardness and chewiness during the refrigerated storage period of tuna chunks. Molina et al. [16] also observed that UV-C radiation (0.80 and 1.60 J/cm^2^) preserved changes on hardness and chewiness in sea bass fillets stored at 4 °C, agreeing with our results. Likewise, Monteiro et al. [27] reported no effect on cohesiveness, springiness, and resilience by UV-C radiation (0.10 and 0.30 J/cm^2^) in tilapia fillets stored under refrigeration. 

## 4. Conclusions

The combination of MAP with 50% CO_2_ and 50% N_2_ and UV-C at 0.30 J/cm^2^ on the reduction of *S. typhimurium* and *E. coli* O157:H7, shelf life, biogenic amines, and oxidative parameters has not been reported in the literature in any food matrix yet. Our results revealed that MAP and MAP/UV were equally effective and ineffective for the reduction of *S. typhimurium* and *E. coli* O157:H7, respectively. On the other hand, UV was the most effective method for the reduction of *S. typhimurium* and *E. coli* O157:H7. UV, MAP, and MAP/UV delayed the growth of TAMC, TAPC, and *Enterobacteriaceae* count, decreased the pH values and ammonia formation prolonging the shelf life of tilapia fillets in 2, 3, and at least 5 days of refrigerated storage. Overall, both methods, MAP and UV alone or in combination, decreased the production of biogenic amines during the refrigerated period, except MAP/UV, which increased histamine levels, and UV showed no effect in putrescine levels. Furthermore, MAP/UV was more effective in preserving texture changes. However, lipid oxidation and discoloration were more pronounced in this treatment. UV did not affect lipid oxidation and was more effective in minimizing discoloration and texture changes than MAP, which increased lipid oxidation in tilapia fillets stored at 4 ± 1 °C for 10 days. Therefore, due to synergistic effects and promising results found for MAP/UV related to longer shelf life and preservation of texture changes, different gas ratios in combination with different UV-C doses should be evaluated to achieve the most significant reduction of potential pathogens and extension of shelf life without compromising the overall quality of tilapia fillets during refrigerated storage. 

## Figures and Tables

**Table 1:** Count of *Salmonella typhimurium* and *Escherichia coli* O157:H7 in tilapia (*Oreochromis niloticus*) fillets non- and treated with ultraviolet radiation (UV-C) and modified atmosphere packaging (MAP).

Treatments ^¥^	Count ^€^	
	*Salmonella typhimurium*	*Escherichia coli* O157:H7
AP	5.98 ± 0.24 ^a^	6.00 ± 0.21 ^a^
VP	5.83 ± 0.21 ^a,b^	5.89 ± 0.29 ^a^
MAP	5.49 ± 0.20 ^b^	5.93 ± 0.27 ^a^
UV	4.86 ± 0.43 ^c^	5.29 ± 0.34 ^b^
MAP/UV	5.48 ± 0.24 ^b^	5.84 ± 0.39 ^a^

Results are expressed as mean ± standard deviation (*n* = 5). ^a,b,c^ Different letters in the same column indicate significant differences (*p* < 0.05) among treatments. ^¥^ AP—air-packed tilapia fillets, VP—vacuum-packed tilapia fillets, MAP—MAP-packed tilapia fillets with 50% CO_2_ and 50% N_2_, UV—UV-C treated tilapia fillets at 0.30 J/cm^2^, and MAP/UV—MAP-packed tilapia fillets with 50% CO_2_ and 50% N_2_ treated with UV-C at 0.30 J/cm^2^. ^€^ Count is expressed in log CFU (colony forming units)/g.

**Table 2:** Bacterial growth parameters of tilapia (*Oreochromis niloticus*) fillets non- and treated with ultraviolet radiation (UV-C) and modified atmosphere packaging (MAP) stored at 4 ± 1 °C for 10 days.

Microorganisms ^£^	Parameters ^€^	Treatments ^¥^				
		AP	VP	MAP	UV	MAP/UV
TAMC	Lag	2.89 ± 0.12 ^c^	3.11 ± 0.18 ^b,c^	3.31 ± 0.04 ^b^	ND	5.59 ± 0.20 ^a^
	GT	0.52 ± 0.02 ^c^	0.46 ± 0.02 ^c^	0.63 ± 0.01 ^b^	0.72 ± 0.02 ^a,b^	0.78 ± 0.09 ^a^
	NC	ND	ND	ND	ND	ND
TAPC	Lag	ND	ND	3.87 ± 0.01	ND	ND
	GT	0.44 ± 0.03 ^d^	0.50 ± 0.01 ^c^	0.40 ± 0.04 ^d^	0.56 ± 0.01 ^b^	0.96 ± 0.09 ^a^
	NC	8.30 ± 0.27	ND	ND	ND	ND
*Enterobacteriaceae*	Lag	ND	ND	2.91 ± 0.04	ND	ND
	GT	0.42 ± 0.01 ^c^	0.42 ± 0.03 ^c^	0.26 ± 0.01 ^d^	0.47 ± 0.02 ^b^	1.01 ± 0.00 ^a^
	NC	ND	ND	7.88 ± 0.06	ND	ND

Results are expressed as mean ± standard deviation (*n* = 10). ^a,b,c,d^ Different letters in the same row within same parameter indicate significant differences (*p* < 0.05) among treatments. ^£^ TAMC—Total aerobic mesophilic count; TAPC—Total aerobic psychrotrophic count. ^€^ Lag—lag phase (h); GT—generation time (time need for bacterial cell duplication in h); NC—number of colonies in the stationary phase in log CFU (colony forming units)/g. ND—Not detectable. ^¥^ AP—air-packed tilapia fillets, VP—vacuum-packed tilapia fillets, MAP—MAP-packed tilapia fillets with 50% CO_2_ and 50% N_2_, UV—UV-C treated tilapia fillets at 0.30 J/cm^2^, and MAP/UV—MAP-packed tilapia fillets with 50% CO_2_ and 50% N_2_ treated with UV-C at 0.30 J/cm^2^.

## Supplementary Materials

The following are available online, File S1: Total aerobic mesophilic count (A), total aerobic psychrotrophic count (B), and *Enterobacteriaceae* count (C) in tilapia (*Oreochromis niloticus*) fillets non- and treated with ultraviolet radiation (UV-C) and modified atmosphere packaging (MAP) stored at 4 ± 1 °C for 10 days, File S2: Biogenic amines, expressed in mg/kg, in tilapia (*Oreochromis niloticus*) fillets non- and treated with ultraviolet radiation (UV-C) and modified atmosphere packaging (MAP) stored at 4 ± 1 °C for 10 days, File S3: Results of pH, ammonia (µg/g) and malondialdehyde (MDA; mg/kg) in tilapia (*Oreochromis niloticus*) fillets non- and treated with ultraviolet radiation (UV-C) and modified atmosphere packaging (MAP) stored at 4 ± 1 °C for 10 days, File S4: L^*^ (lightness), a^*^ (redness), b^*^ (yellowness) parameters and total color difference (∆E) in tilapia (*Oreochromis niloticus*) fillets non- and treated with ultraviolet radiation (UV-C) and modified atmosphere packaging (MAP) stored at 4 ± 1 °C for 10 days, File S5: Results of hardness (Newton - N), chewiness (N × mm), cohesiveness (ratio), springiness (ratio) and resilience (ratio) in tilapia (*Oreochromis niloticus*) fillets non- and treated with ultraviolet radiation (UV-C) and modified atmosphere packaging (MAP) stored at 4 ± 1 °C for 10 days.
