# Supplementary material for: Combined Effect of Modified Atmosphere Packaging and UV-C Radiation on Pathogens Reduction, Biogenic Amines, and Shelf Life of Refrigerated Tilapia (Oreochromis niloticus) Fillets
Source: Molecules. 2020 Jul 15;25(14):3222. doi: 10.3390/molecules25143222 (PMC7397001; doi:10.3390/molecules25143222)
Supplement: Supplementary file 1 [file molecules-25-03222-s001.pdf]

molecules

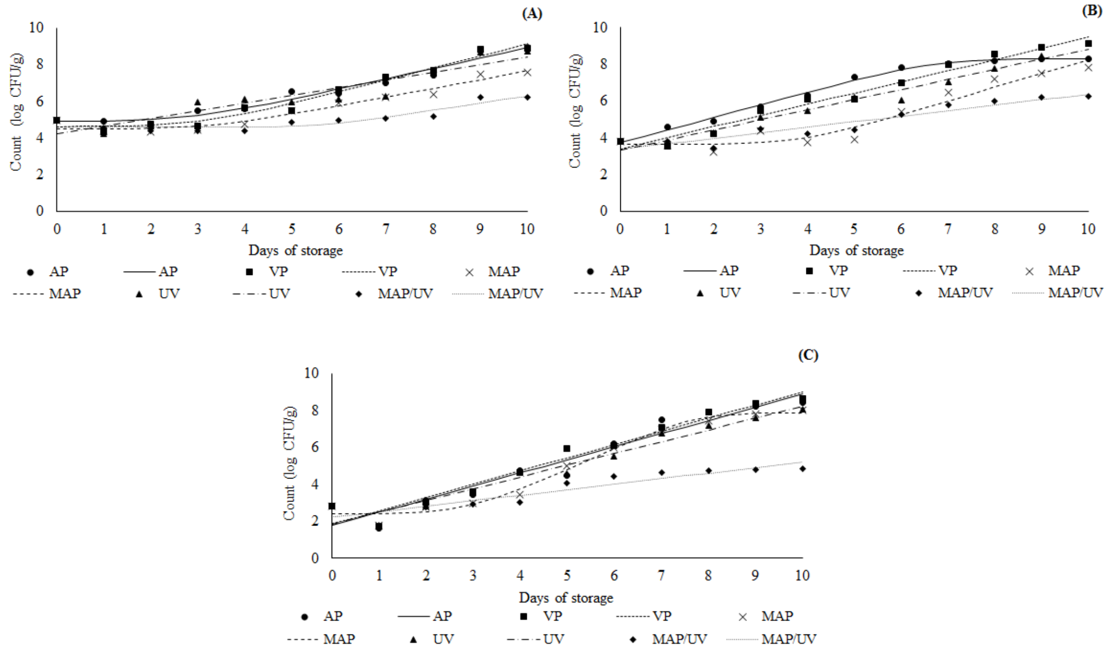

Supplementary File S1. Total aerobic mesophilic count (A), total aerobic psychrotrophic count (B), and *Enterobacteriaceae* count (C) in tilapia (*Oreochromis niloticus*) fillets non- and treated with ultraviolet radiation (UV-C) and modified atmosphere packaging (MAP) stored at 4 ± 1 °C for 10 days. Results are expressed as the mean of log CFU (colony forming units)/g ± standard deviation (n = 10). Symbols indicate the real average values and lines represent the fitted values by primary predictive model designed by Baranyi and Roberts [34]. AP – air-packed tilapia fillets, VP – vacuum-packed tilapia fillets, MAP – MAP-packed tilapia fillets with 50% CO_2 and 50% N_2, UV – UV-C treated tilapia fillets at 0.30 J/cm^2, and MAP/UV – MAP-packed tilapia fillets with 50% CO_2 and 50% N_2 treated with UV-C at 0.30 J/cm^2

molecules

MDPI

Supplementary File S2. Biogenic amines, expressed in mg/kg, in tilapia (*Oreochromis niloticus*) fillets non- and treated with ultraviolet radiation (UV-C) and modified atmosphere packaging (MAP) stored at 4 ± 1 °C for 10 days.

Parameters	Treatments$^{¥}$	Days of storage									
		1	2	3	4	5	6	7	8	9	10
**Histamine**	AP	0.05±0.01^{c}	0.04±0.00^{c}	0.07±0.00^{c}	0.16±0.01^{b}	0.17±0.02^{b}	0.22±0.03^{a}	0.17±0.01^{b}	0.17±0.02^{b}	0.05±0.00^{c}	0.04±0.00^{c}
	VP	0.06±0.01^{e}	0.17±0.01^{b}	0.14±0.00^{c}	0.24±0.01^{a}	0.11±0.01^{d}	0.14±0.01^{c}	0.06±0.00^{e}	0.06±0.00^{e}	0.06±0.01^{e}	0.06±0.00^{e}
	MAP	0.00±0.00^{e}	0.05±0.00^{cd}	0.07±0.01^{bc}	0.07±0.00^{b}	0.05±0.00^{d}	0.08±0.01^{b}	0.16±0.01^{a}	0.17±0.01^{a}	0.05±0.01^{d}	0.05±0.00^{d}
	UV	0.05±0.00^{fg}	0.13±0.00^{bc}	0.12±0.00^{cd}	0.12±0.00^{cd}	0.14±0.01^{ab}	0.15±0.01^{a}	0.12±0.00^{d}	0.09±0.00^{e}	0.04±0.00^{g}	0.06±0.01^{f}
	MAP/UV	0.11±0.01^{e}	0.34±0.00^{c}	0.47±0.03^{b}	0.79±0.00^{a}	0.22±0.00^{d}	0.08±0.00^{ef}	0.07±0.00^{f}	0.08±0.01^{f}	0.09±0.01^{ef}	0.08±0.00^{ef}
Tyramine	AP	0.07±0.00^{e}	0.04±0.00^{f}	0.04±0.00^{f}	0.08±0.00^{e}	0.22±0.00^{d}	0.34±0.00^{c}	0.46±0.00^{a}	0.44±0.00^{b}	0.33±0.01^{c}	0.04±0.00^{f}
	VP	0.04±0.00^{c}	0.04±0.00^{c}	0.16±0.00^{b}	0.32±0.00^{a}	0.04±0.00^{c}	0.04±0.00^{c}	0.04±0.00^{c}	0.03±0.00^{d}	0.04±0.00^{c}	0.04±0.00^{c}
	MAP	0.04±0.00^{d}	0.04±0.00^{d}	0.04±0.00^{d}	0.32±0.01^{a}	0.29±0.00^{b}	0.32±0.00^{a}	0.22±0.00^{c}	0.02±0.00^{e}	0.04±0.00^{d}	0.04±0.00^{d}
	UV	0.04±0.00^{a}	0.04±0.00^{a}	0.04±0.00^{a}	0.04±0.00^{a}	0.04±0.00^{a}	0.04±0.00^{a}	0.04±0.00^{a}	0.04±0.00^{a}	0.04±0.00^{a}	0.04±0.00^{a}
	MAP/UV	0.04±0.00^{b}	0.04±0.00^{ab}	0.04±0.00^{b}	0.05±0.00^{a}	0.03±0.00^{c}	0.04±0.00^{b}	0.04±0.00^{b}	0.04±0.00^{b}	0.04±0.00^{b}	0.04±0.00^{b}
Cadaverine	AP	0.01±0.00^{e}	0.01±0.00^{e}	0.04±0.00^{c}	0.03±0.00^{de}	0.04±0.00^{cd}	0.04±0.00^{cd}	0.04±0.00^{cd}	0.06±0.00^{b}	0.07±0.01^{b}	0.19±0.01^{a}
	VP	0.00±0.00^{e}	0.02±0.00^{d}	0.03±0.00^{c}	0.02±0.00^{d}	0.02±0.00^{d}	0.01±0.00^{de}	0.07±0.01^{b}	0.07±0.00^{b}	0.11±0.00^{a}	0.12±0.00^{a}
	MAP	0.00±0.00^{f}	0.00±0.00^{f}	0.01±0.00^{e}	0.02±0.00^{d}	0.04±0.00^{c}	0.02±0.00^{d}	0.03±0.00^{c}	0.08±0.00^{a}	0.07±0.00^{b}	0.07±0.00^{b}
	UV	0.00±0.00^{fg}	0.00±0.00^{g}	0.01±0.00^{f}	0.01±0.00^{fg}	0.01±0.00^{e}	0.02±0.00^{d}	0.06±0.00^{b}	0.07±0.00^{a}	0.05±0.00^{c}	0.05±0.00^{c}
	MAP/UV	0.02±0.00^{e}	0.02±0.00^{e}	0.01±0.00^{e}	0.04±0.00^{d}	0.05±0.00^{c}	0.02±0.00^{e}	0.02±0.00^{e}	0.02±0.00^{e}	0.10±0.00^{b}	0.16±0.00^{a}
Putrescine	AP	0.01±0.00^{e}	0.01±0.00^{e}	0.01±0.00^{e}	0.01±0.00^{e}	0.06±0.00^{c}	0.07±0.00^{c}	0.07±0.00^{c}	0.04±0.00^{d}	0.11±0.00^{b}	0.14±0.00^{a}
	VP	0.01±0.00^{d}	0.01±0.00^{d}	0.01±0.00^{d}	0.01±0.00^{d}	0.01±0.00^{d}	0.01±0.00^{d}	0.13±0.00^{c}	0.17±0.00^{b}	0.30±0.00^{a}	0.14±0.00^{c}
	MAP	0.00±0.00^{g}	0.01±0.00^{f}	0.01±0.00^{g}	0.01±0.00^{g}	0.01±0.00^{e}	0.01±0.00^{d}	0.01±0.00^{e}	0.03±0.00^{c}	0.14±0.00^{b}	0.18±0.00^{a}
	UV	0.01±0.00^{de}	0.00±0.00^{e}	0.01±0.00^{de}	0.01±0.00^{de}	0.01±0.00^{de}	0.01±0.00^{de}	0.01±0.00^{d}	0.14±0.00^{c}	0.22±0.01^{a}	0.15±0.00^{b}
	MAP/UV	0.05±0.00^{a}	0.02±0.00^{c}	0.03±0.00^{b}	0.04±0.00^{b}	0.01±0.00^{de}	0.02±0.00^{d}	0.01±0.00^{e}	0.01±0.00^{e}	0.01±0.00^{e}	0.03±0.00^{b}
Spermidine	AP	0.01±0.00^{de}	0.01±0.00^{cd}	0.01±0.00^{cd}	0.01±0.00^{e}	0.01±0.00^{de}	0.01±0.00^{e}	0.01±0.00^{c}	0.02±0.00^{b}	0.02±0.00^{b}	0.03±0.00^{a}
	VP	0.01±0.00^{ab}	0.01±0.00^{c}	0.01±0.00^{a}	0.01±0.00^{bc}	0.01±0.00^{c}	0.01±0.00^{c}	0.01±0.00^{a}	0.01±0.00^{bc}	0.01±0.00^{bc}	0.01±0.00^{bc}
	MAP	0.01±0.00^{bcd}	0.01±0.00^{abc}	0.01±0.00^{e}	0.01±0.00^{bcd}	0.01±0.00^{de}	0.01±0.00^{cd}	0.01±0.00^{a}	0.01±0.00^{ab}	0.01±0.00^{de}	0.01±0.00^{bcd}
	UV	0.01±0.00^{abcd}	0.01±0.00^{bcd}	0.01±0.00^{bcd}	0.01±0.00^{d}	0.01±0.00^{bcd}	0.01±0.00^{abcd}	0.01±0.00^{ab}	0.01±0.00^{abc}	0.01±0.00^{a}	0.01±0.00^{cd}
	MAP/UV	0.01±0.00^{ab}	0.01±0.00^{abc}	0.01±0.00^{abc}	0.01±0.00^{bc}	0.01±0.00^{ab}	0.01±0.00^{c}	0.01±0.00^{abc}	0.01±0.00^{a}	0.01±0.00^{abc}	0.01±0.00^{a}
Spermine	AP	0.06±0.01^{c}	0.06±0.00^{cd}	0.06±0.00^{cd}	0.04±0.00^{e}	0.06±0.00^{c}	0.07±0.00^{bc}	0.08±0.01^{a}	0.06±0.00^{c}	0.07±0.00^{ab}	0.04±0.00^{de}
	VP	0.07±0.01^{a}	0.05±0.00^{cd}	0.04±0.00^{de}	0.04±0.01^{de}	0.04±0.00^{e}	0.03±0.00^{e}	0.04±0.00^{de}	0.04±0.00^{de}	0.06±0.01^{ab}	0.06±0.00^{bc}

MAP	0.08±0.01[a]	0.04±0.00[def]	0.05±0.00[de]	0.04±0.00[efg]	0.03±0.00[g]	0.03±0.00[fg]	0.06±0.00[cd]	0.06±0.00[cd]	0.06±0.00[bc]	0.07±0.00[ab]
UV	0.06±0.01[a]	0.03±0.00[cd]	0.03±0.00[d]	0.03±0.00[d]	0.03±0.00[bcd]	0.04±0.00[bc]	0.06±0.00[a]	0.04±0.00[b]	0.06±0.00[a]	0.05±0.00[a]
MAP/UV	0.07±0.00[a]	0.05±0.00[bc]	0.05±0.00[bc]	0.04±0.00[cd]	0.05±0.00[bc]	0.04±0.00[cd]	0.03±0.00[d]	0.05±0.00[bc]	0.05±0.00[bc]	0.05±0.01[b]

Results are expressed as mean ± standard deviation (n = 10). [a,b,c,d,e,f,g]Different letters in the same row within same treatment indicate significant differences ($p < 0.05$) among days of storage. [¥]AP – air-packed tilapia fillets, VP – vacuum-packed tilapia fillets, MAP – MAP-packed tilapia fillets with 50% CO_2 and 50% N_2, UV – UV-C treated tilapia fillets at 0.30 J/cm^2, and MAP/UV – MAP-packed tilapia fillets with 50% CO_2 and 50% N_2 treated with UV-C at 0.30 J/cm^2.

Supplementary File S3. Results of pH, ammonia (μg/g) and malondialdehyde (MDA; mg/kg) in tilapia (*Oreochromis niloticus*) fillets non- and treated with ultraviolet radiation (UV-C) and modified atmosphere packaging (MAP) stored at 4 ± 1 °C for 10 days.

**Parameters**	Treatments[¥]	Days of storage									
		1	2	3	4	5	6	7	8	9	10
	AP	6.23±0.01^{b}	6.29±0.01^{a}	6.20±0.01^{bc}	6.19±0.01^{bc}	6.20±0.01^{bc}	6.15±0.01^{cd}	6.12±0.01^{d}	6.17±0.03^{bcd}	6.05±0.01^{e}	5.78±0.06^{f}
	VP	6.20±0.01^{ab}	6.25±0.02^{a}	6.20±0.02^{ab}	6.19±0.01^{ab}	6.03±0.06^{c}	6.16±0.01^{b}	5.59±0.03^{e}	6.04±0.01^{c}	5.98±0.02^{d}	6.07±0.01^{c}
**pH**	MAP	6.27±0.01^{a}	6.19±0.02^{b}	6.21±0.02^{b}	6.19±0.01^{b}	6.11±0.01^{c}	5.96±0.01^{e}	5.79±0.02^{f}	5.75±0.02^{f}	6.06±0.01^{d}	6.06±0.02^{d}
	UV	6.24±0.01^{b}	6.30±0.02^{a}	6.16±0.01^{c}	6.30±0.01^{a}	6.12±0.01^{c}	6.16±0.01^{c}	5.92±0.01^{d}	5.84±0.01^{e}	5.67±0.04^{f}	5.94±0.01^{d}
	MAP/UV	6.20±0.00^{ab}	6.23±0.02^{a}	6.14±0.01^{c}	6.15±0.01^{bc}	6.00±0.01^{de}	6.15±0.05^{bc}	5.95±0.02^{e}	5.79±0.01^{f}	5.74±0.01^{g}	6.02±0.01^{d}
	AP	0.70±0.04^{e}	0.74±0.04^{e}	1.18±0.10^{cd}	0.97±0.02^{d}	1.09±0.05^{cd}	1.03±0.04^{d}	1.01±0.06^{d}	1.87±0.03^{a}	1.25±0.08^{c}	1.52±0.03^{b}
	VP	0.78±0.06^{d}	1.02±0.04^{cd}	1.03±0.06^{c}	1.03±0.07^{c}	1.02±0.01^{cd}	1.03±0.08^{c}	1.19±0.04^{bc}	1.31±0.10^{ab}	1.25±0.07^{abc}	1.46±0.02^{a}
Ammonia	MAP	0.78±0.05^{ef}	0.57±0.05^{f}	1.02±0.07^{cd}	0.93±0.03^{de}	0.93±0.07^{de}	0.89±0.04^{de}	0.97±0.06^{cde}	1.17±0.07^{bc}	1.32±0.10^{ab}	1.51±0.00^{a}
	UV	0.61±0.04^{d}	0.77±0.06^{d}	1.14±0.02^{abc}	1.11±0.07^{abc}	1.02±0.04^{c}	1.01±0.03^{c}	1.11±0.04^{abc}	1.07±0.06^{bc}	1.28±0.11^{ab}	1.30±0.00^{a}
	MAP/UV	0.84±0.05^{de}	0.87±0.05^{cde}	0.81±0.07^{e}	0.96±0.09^{cde}	0.95±0.06^{cde}	1.10±0.01^{bc}	0.99±0.00^{cde}	1.08±0.06^{bcd}	1.25±0.10^{ab}	1.35±0.06^{a}
	AP	0.04±0.00^{e}	0.04±0.00^{e}	0.01±0.00^{f}	0.03±0.00^{e}	0.07±0.00^{d}	0.01±0.00^{f}	0.22±0.01^{a}	0.08±0.01^{d}	0.11±0.00^{c}	0.19±0.01^{b}
	VP	0.02±0.00^{e}	0.04±0.00^{c}	0.04±0.00^{c}	0.06±0.00^{b}	0.03±0.00^{d}	0.04±0.00^{c}	0.19±0.00^{a}	0.02±0.00^{e}	0.02±0.00^{e}	0.02±0.00^{e}
MDA	MAP	0.02±0.00^{g}	0.03±0.00^{e}	0.02±0.00^{f}	0.05±0.00^{d}	0.06±0.00^{c}	0.03±0.00^{f}	0.02±0.00^{f}	0.38±0.00^{b}	0.41±0.00^{a}	0.03±0.00^{e}
	UV	0.05±0.00^{c}	0.02±0.00^{f}	0.03±0.00^{e}	0.03±0.00^{de}	0.04±0.00^{d}	0.06±0.00^{c}	0.02±0.00^{f}	0.12±0.00^{a}	0.05±0.00^{c}	0.07±0.00^{b}
	MAP/UV	0.04±0.00^{h}	0.05±0.00^{gh}	0.10±0.00^{fg}	0.15±0.01^{ef}	0.16±0.00^{e}	0.26±0.00^{d}	0.32±0.00^{c}	0.75±0.04^{b}	0.90±0.01^{a}	0.11±0.01^{ef}

Results are expressed as mean ± standard deviation (n = 10). [a,b,c,d,e,f,g,h]Different letters in the same row within same treatment indicate significant differences ($p < 0.05$) among days of storage. [¥]AP – air-packed tilapia fillets, VP – vacuum-packed tilapia fillets, MAP – MAP-packed tilapia fillets with 50% CO_2 and 50% N_2, UV – UV-C treated tilapia fillets at 0.30 J/cm^2, and MAP/UV – MAP-packed tilapia fillets with 50% CO_2 and 50% N_2 treated with UV-C at 0.30 J/cm^2.

molecules

MDPI

Supplementary File S4. L* (lightness), a* (redness), b* (yellowness) parameters and total color difference (ΔE) in tilapia (*Oreochromis niloticus*) fillets non- and treated with ultraviolet radiation (UV-C) and modified atmosphere packaging (MAP) stored at 4 ± 1 °C for 10 days.

Parameters	Treatments¥	Days of storage									
		1	2	3	4	5	6	7	8	9	10
L*	AP	59.50±2.17^{ab}	58.95±2.96^{ab}	58.40±1.61^{ab}	59.64±3.56^{ab}	60.53±1.61^{ab}	56.82±1.78^{b}	60.06±5.75^{ab}	59.12±2.91^{ab}	63.91±2.94^{a}	64.11±2.59^{a}
	VP	57.98±2.22^{cde}	58.48±2.39^{bcde}	61.90±2.90^{ab}	57.20±1.63^{de}	58.91±1.39^{abcde}	56.53±0.96^{e}	62.12±2.37^{a}	59.44±1.22^{abcde}	60.58±1.21^{abcd}	60.93±0.77^{abc}
	MAP	55.55±2.18^{b}	60.89±3.76^{ab}	63.16±2.66^{a}	58.74±2.69^{ab}	62.94±3.59^{a}	62.00±1.56^{a}	58.66±1.13^{ab}	60.88±4.67^{ab}	64.46±5.11^{a}	59.87±3.53^{ab}
	UV	59.99±2.28^{bcd}	58.13±1.53^{cd}	61.36±1.35^{abc}	60.79±3.16^{abcd}	61.35±3.10^{abc}	59.79±3.44^{bcd}	56.87±1.90^{d}	58.21±1.58^{cd}	64.89±2.02^{a}	63.97±1.42^{ab}
	MAP/UV	59.61±1.78^{ab}	60.48±3.16^{ab}	60.15±1.88^{ab}	64.01±4.00^{a}	64.67±3.88^{a}	58.67±2.48^{b}	59.81±2.67^{ab}	60.50±2.97^{ab}	64.44±1.80^{a}	63.81±1.77^{ab}
a*	AP	0.91±0.06^{a}	-1.15±0.11^{cd}	-2.47±0.28^{g}	-2.03±0.11^{f}	-1.20±0.13^{cd}	-1.79±0.09^{ef}	-1.03±0.05^{bc}	-0.73±0.05^{b}	-1.00±0.12^{bc}	-1.47±0.12^{de}
	VP	0.25±0.03^{a}	-0.56±0.06^{b}	-0.65±0.03^{bc}	-1.02±0.07^{de}	-1.19±0.15^{e}	-1.27±0.04^{e}	-1.82±0.14^{f}	-0.76±0.13^{bcd}	-0.86±0.11^{cd}	-0.90±0.07^{cd}
	MAP	-1.16±0.17^{b}	-0.40±0.04^{a}	-2.57±0.03^{c}	-1.42±0.11^{b}	-1.45±0.16^{b}	-2.49±0.13^{c}	-2.53±0.08^{c}	-1.47±0.18^{b}	-2.41±0.05^{c}	-3.96±0.03^{d}
	UV	-1.24±0.21^{bc}	-1.46±0.13^{cd}	-0.83±0.03^{a}	-1.30±0.03^{bc}	-1.24±0.03^{bc}	-2.12±0.11^{e}	-1.64±0.03^{d}	-0.81±0.11^{a}	-1.00±0.15^{ab}	-2.66±0.04^{f}
	MAP/UV	-2.21±0.14^{cd}	-1.64±0.12^{b}	-0.55±0.05^{a}	-1.66±0.21^{b}	-2.04±0.16^{bcd}	-2.01±0.04^{bcd}	-1.97±0.07^{bc}	-2.86±0.49^{e}	-1.58±0.10^{b}	-2.52±0.03^{e}
b*	AP	9.20±0.87^{bc}	8.51±0.36^{c}	9.43±0.52^{bc}	11.13±0.79^{a}	9.13±0.78^{bc}	9.24±0.40^{bc}	12.16±0.20^{a}	9.40±0.81^{bc}	11.86±0.67^{a}	10.67±0.73^{ab}
	VP	7.28±0.42^{cd}	9.55±0.52^{b}	8.36±0.08^{bcd}	8.79±0.74^{bc}	7.09±0.06^{d}	7.85±0.55^{cd}	11.34±1.01^{a}	7.81±0.54^{cd}	11.55±0.99^{a}	8.11±0.57^{bcd}
	MAP	8.11±1.11^{d}	12.56±0.88^{a}	10.51±1.35^{abc}	10.10±0.52^{bcd}	12.14±0.57^{ab}	10.74±0.51^{abc}	9.22±0.56^{cd}	10.80±0.85^{abc}	12.54±1.11^{a}	11.40±0.93^{ab}
	UV	8.50±0.69^{cd}	9.94±0.60^{b}	8.45±0.61^{cd}	8.74±0.20^{c}	7.48±0.54^{d}	8.38±0.07^{cd}	11.27±0.69^{a}	7.60±0.16^{cd}	11.34±0.04^{a}	8.39±0.63^{cd}
	MAP/UV	10.45±0.35^{c}	12.19±0.92^{b}	11.59±0.53^{bc}	8.57±0.43^{d}	11.49±0.51^{bc}	11.26±0.62^{bc}	12.45±0.70^{b}	12.55±0.75^{b}	14.42±0.39^{a}	11.23±0.90^{bc}

Parameters	Treatments	Total color difference
Δ_{10-0}€	AP	5.46±0.72^{b}
	VP	4.15±0.14^{c}
	MAP	6.79±0.62^{a}
	UV	4.24±0.30^{c}
	MAP/UV	6.81±0.83^{a}

Results are expressed as mean ± standard deviation (n = 10). [a,b,c,d,e,f,g]Different letters in the same row within same treatment indicate significant differences ($p < 0.05$) among days of storage. ¥AP – air-packed tilapia fillets, VP – vacuum-packed tilapia fillets, MAP – MAP-packed tilapia fillets with 50% CO_2 and 50% N_2, UV – UV-C treated tilapia fillets at 0.30 J/cm², and MAP/UV – MAP-packed tilapia fillets with 50% CO_2 and 50% N_2 treated with UV-C at 0.30 J/cm². €ΔE10-0 - Total color difference between days 10 and 0 of refrigerated storage of each treatment. [a,b,c]Different letters in the column indicate significant differences ($p < 0.05$) among treatments.

Supplementary File S5. Results of hardness (Newton - N), chewiness (N × mm), cohesiveness (ratio), springiness (ratio) and resilience (ratio) in tilapia (*Oreochromis niloticus*) fillets non- and treated with ultraviolet radiation (UV-C) and modified atmosphere packaging (MAP) stored at 4 ± 1 °C for 10 days.

**Parameters**	Treatments$^{¥}$	Days of storage									
		1	2	3	4	5	6	7	8	9	10
**Hardness**	AP	22.69±2.93^{a}	23.07±2.51^{a}	21.17±2.40^{a}	12.23±1.46^{bc}	13.29±2.04^{bc}	15.07±2.43^{b}	9.10±0.96^{c}	12.10±0.62^{bc}	14.63±0.08^{b}	9.73±1.31^{bc}
	VP	16.37±0.66^{a}	16.17±0.67^{a}	15.60±0.35^{ab}	15.49±0.82^{ab}	15.52±0.46^{ab}	15.40±0.03^{ab}	14.06±0.40^{bc}	14.16±0.34^{bc}	13.32±0.75^{c}	13.15±0.82^{c}
	MAP	25.67±0.55^{a}	25.77±0.39^{a}	25.25±2.15^{a}	21.19±0.15^{b}	19.86±1.58^{bc}	16.99±0.43^{cd}	15.04±1.78^{d}	16.53±0.83^{d}	15.40±0.87^{d}	13.89±0.86^{d}
	UV	25.62±1.10^{a}	24.26±1.19^{ab}	23.63±1.48^{abc}	22.40±0.91^{abc}	20.69±1.05^{cd}	21.36±1.13^{bcd}	21.19±1.87^{bcd}	18.55±1.24^{de}	18.08±0.60^{de}	17.09±1.37^{e}
	MAP/UV	23.35±1.79^{ab}	22.81±1.29^{ab}	24.33±4.17^{ab}	25.60±1.41^{a}	24.84±1.64^{ab}	23.52±0.80^{ab}	22.30±1.27^{ab}	22.99±1.23^{ab}	22.33±2.19^{ab}	19.64±2.14^{b}
Chewiness	AP	7.54±0.81^{a}	5.75±0.50^{b}	6.10±0.30^{b}	3.82±0.49^{c}	2.53±0.07^{d}	2.67±0.27^{d}	2.34±0.20^{d}	3.19±0.26^{cd}	2.25±0.16^{d}	2.94±0.13^{cd}
	VP	5.20±0.34^{a}	4.62±0.69^{ab}	3.84±0.34^{bcde}	4.10±0.47^{bc}	2.96±0.03^{ef}	4.03±0.12^{bcd}	4.20±0.01^{b}	3.21±0.07^{cdef}	3.16±0.23^{def}	2.81±0.12^{f}
	MAP	7.07±0.50^{a}	7.32±0.91^{a}	7.12±0.84^{a}	6.34±0.48^{ab}	5.62±0.14^{b}	2.45±0.29^{c}	2.86±0.37^{c}	1.66±0.17^{c}	1.84±0.07^{c}	1.86±0.05^{c}
	UV	6.68±0.12^{ab}	7.04±0.13^{a}	7.51±0.44^{a}	6.96±0.60^{ab}	4.77±0.34^{d}	5.36±0.51^{cd}	6.00±0.37^{bc}	5.50±0.19^{cd}	3.72±0.31^{e}	2.87±0.25^{e}
	MAP/UV	8.54±0.84^{a}	7.88±0.26^{a}	8.49±0.41^{a}	6.06±0.28^{bc}	6.69±0.32^{b}	6.03±0.41^{bc}	5.19±0.49^{c}	5.41±0.24^{c}	5.04±0.24^{c}	3.01±0.16^{d}
Cohesiveness	AP	0.54±0.03^{a}	0.52±0.05^{a}	0.52±0.01^{a}	0.52±0.01^{a}	0.50±0.04^{a}	0.50±0.01^{a}	0.52±0.06^{a}	0.49±0.04^{a}	0.51±0.04^{a}	0.52±0.01^{a}
	VP	0.51±0.04^{a}	0.55±0.03^{a}	0.54±0.02^{a}	0.52±0.03^{a}	0.49±0.02^{a}	0.50±0.01^{a}	0.52±0.06^{a}	0.50±0.02^{a}	0.52±0.02^{a}	0.51±0.02^{a}
	MAP	0.49±0.02^{a}	0.51±0.02^{a}	0.50±.002^{a}	0.52±0.05^{a}	0.49±0.02^{a}	0.52±0.03^{a}	0.52±0.03^{a}	0.51±0.04^{a}	0.50±0.01^{a}	0.49±0.05^{a}
	UV	0.50±0.03^{a}	0.50±0.04^{a}	0.49±0.01^{a}	0.53±0.01^{a}	0.53±0.02^{a}	0.50±0.02^{a}	0.51±0.00^{a}	0.50±0.04^{a}	0.49±0.04^{a}	0.51±0.02^{a}
	MAP/UV	0.50±0.07^{a}	0.52±0.02^{a}	0.52±0.04^{a}	0.54±0.01^{a}	0.54±0.01^{a}	0.53±0.03^{a}	0.52±0.02^{a}	0.50±0.05^{a}	0.51±0.04^{a}	0.51±0.02^{a}
Springiness	AP	0.53±0.01^{a}	0.53±0.01^{a}	0.56±0.01^{a}	0.54±0.02^{a}	0.54±0.01^{a}	0.53±0.01^{a}	0.54±0.05^{a}	0.52±0.03^{a}	0.54±0.02^{a}	0.55±0.02^{a}
	VP	0.56±0.02^{a}	0.56±0.04^{a}	0.56±0.04^{a}	0.52±0.02^{a}	0.50±0.02^{a}	0.55±0.02^{a}	0.56±0.03^{a}	0.56±0.03^{a}	0.52±0.02^{a}	0.53±0.02^{a}
	MAP	0.53±0.04^{a}	0.51±0.08^{a}	0.56±0.03^{a}	0.54±0.01^{a}	0.55±0.03^{a}	0.54±0.02^{a}	0.52±0.05^{a}	0.53±0.02^{a}	0.53±0.02^{a}	0.53±0.01^{a}
	UV	0.52±0.02^{a}	0.51±0.02^{a}	0.54±0.02^{a}	0.54±0.01^{a}	0.53±0.01^{a}	0.54±0.03^{a}	0.52±0.01^{a}	0.53±0.01^{a}	0.51±0.01^{a}	0.54±0.01^{a}
	MAP/UV	0.57±0.03^{a}	0.57±0.01^{a}	0.56±0.03^{a}	0.52±0.03^{a}	0.58±0.00^{a}	0.52±0.06^{a}	0.53±0.01^{a}	0.52±0.03^{a}	0.51±0.02^{a}	0.53±0.03^{a}
Resilience	AP	0.14±0.01^{a}	0.14±0.01^{a}	0.14±0.01^{a}	0.14±0.01^{a}	0.15±0.01^{a}	0.15±0.01^{a}	0.16±0.01^{a}	0.15±0.01^{a}	0.15±0.01^{a}	0.15±0.01^{a}
	VP	0.14±0.01^{a}	0.14±0.01^{a}	0.14±0.01^{a}	0.14±0.01^{a}	0.15±0.01^{a}	0.15±0.01^{a}	0.15±0.01^{a}	0.16±0.01^{a}	0.16±0.02^{a}	0.14±0.01^{a}
	MAP	0.13±0.01^{a}	0.14±0.01^{a}	0.13±0.01^{a}	0.14±0.01^{a}	0.15±0.01^{a}	0.15±0.01^{a}	0.15±0.01^{a}	0.15±0.01^{a}	0.16±0.01^{a}	0.14±0.02^{a}
	UV	0.15±0.01^{a}	0.15±0.00^{a}	0.15±0.01^{a}	0.14±0.01^{a}	0.15±0.01^{a}	0.14±0.01^{a}	0.15±0.01^{a}	0.14±0.00^{a}	0.15±0.00^{a}	0.15±0.02^{a}
	MAP/UV	0.14±0.01^{a}	0.14±0.02^{a}	0.14±0.01^{a}	0.15±0.01^{a}	0.15±0.01^{a}	0.15±0.01^{a}	0.16±0.00^{a}	0.14±0.01^{a}	0.15±0.01^{a}	0.15±0.01^{a}

Results are expressed as mean ± standard deviation (n = 10). ^{a,b,c,d,e,f}Different letters in the same row within same treatment indicate significant differences ($p < 0.05$) among days of storage. $^{¥}$AP – air-packed tilapia fillets, VP – vacuum-packed tilapia fillets, MAP – MAP-packed tilapia fillets with 50% CO_2 and 50% N_2, UV – UV-C treated tilapia fillets at 0.30 J/cm^2, and MAP/UV – MAP-packed tilapia fillets with 50% CO_2 and 50% N_2 treated with UV-C at 0.30 J/cm^2.
